# Myasthenic crises are associated with negative long-term outcomes in myasthenia gravis

**DOI:** 10.1007/s00415-024-12478-y

**Published:** 2024-06-05

**Authors:** Anna Mück, Steffen Pfeuffer, Lara Mir, Sonja Genau, Julia Emde, Linus Olbricht, Omar A. Omar, Franz Blaes, Christoph Best, Hagen B. Huttner, Heidrun H. Krämer

**Affiliations:** 1 Department of Neurology, Justus Liebig University, Klinikstrasse 33, 35392 Giessen, Germany; 2 Department of Neurology, Klinikum Oberberg, Gummersbach, Germany; 3 Department of Neurology, Philipps-University, Marburg, Germany

**Keywords:** Myasthenia gravis, Myasthenic exacerbation, Disease outcomes, Real-world study

## Introduction

Myasthenia gravis (MG) is a rare antibody-mediated disorder of the neuromuscular junction with an annual incidence ranging from 0.5 to 3 per 100.000 patients [4]. A fluctuating, activity-dependent weakness predominantly affects ocular, bulbar, and/or limb and trunk skeletal muscles. Treatment comprises symptomatic (cholinesterase inhibitors) as well as immunosuppressive treatment.

Besides chronic daytime-dependent aggravation of symptoms, MG can present with acute myasthenic exacerbation (ME) comprising progressive muscle weakness, dysphagia, diplopia, and respiratory deterioration. It can further deteriorate a life-threatening myasthenic crisis (MC) requiring ICU treatment and mechanic ventilation [5].

Rescue treatment includes removal of pathogenic autoantibodies using therapeutic apheresis (plasma exchange (PLEX) or immunoadsorption) or administration of intravenous immunoglobulins (IVIg). However, a substantial proportion of MC patients requires intubation and mechanical ventilation [6].

Common triggers of ME/MC are infections, especially bacterial or viral upper airway infections, such as pneumonia or bronchitis [15]. Moreover, several drugs are known to worsen MG and subsequently cause ME/MC [11]. On the other hand, dose reduction of cholinesterase inhibitors or immunosuppressive agents (e.g., due to side effects or insufficient response) can result in ME/MC as well. However, in up to 40% of the patients, no obvious acute trigger can be identified [13].

Apart from such acute triggers, generalized forms of MG are more prone to development of ME/MC as well as patients with higher baseline impairment [15]. Presence of a thymoma is associated with higher risk of ME/MC as well given that patients with thymoma are characterized by more severe disease courses in general [15].

The impact of ME/MC on the long-term disease course of MG remains poorly understood. Thus, we evaluated the disease courses of MG patients with and without ME in our retrospective tertiary center cohort.

## Methods

### Study design and participants

This is a post hoc analysis of a longitudinal monocentric cohort of MG patients. The diagnosis of MG was established in accordance with national guidelines including repetitive nerve stimulation and antibody testing.

For the current study, we selected patients according to the following criteria: (1) admission between January 2022 and August 2023; (2) presence of QMG scores every 3 months after myasthenic exacerbation (ME) or in patients without ME, over a period of ≥ 1 year after first visit of the study center; (3) exclusive treatment of the MG in our center. In-depth medical chart reviews were performed to validate presence of ME.

Data from selected patients were complemented by retrospectively evaluated data (immunotherapy at ME onset, hospitalization during ME/MC, presence of concomitant autoimmunity, disease duration until ME/MC).

Patients were stratified according to presence (ME^+^) or absence (ME^−^) of myasthenic exacerbation. ME^+^ patients were further subdivided in individuals with myasthenic exacerbation (ME) and manifest myasthenic crisis (MC). ME was defined as a significant worsening of clinical status (increase of at least three points in QMG) with need of hospitalization and rescue therapy (plasma separation or IVIg). MC was specified as additional respiratory deterioration with need for invasive or non-invasive ventilation. To simplify wording, we will use the term ME for both, MC and ME in the further course.

In ME^+^ patients, disease duration was defined as time of diagnosis of MG until ME. In ME^−^ patients, disease duration was determined as time of first clinical symptoms until first visit of our center. Besides clinical data from ME onset (“ME”), we included data from the last visit prior to ME (“baseline 2”) and the first visit after ME (“baseline 3”) in ME^+^ patients as well as data three months prior (“baseline 1”) and after the ME (“3mos”). This setup was chosen to allow comparison to ME^−^ patients, in whom “baseline 3” was defined as first visit in our center, followed by trimestrial visits (“mos 3; 6; 9; 12”) in both subgroups.

This study was approved by the local Ethics Committee of the Justus-Liebig-University Giessen (189/22 and 50/23). Anonymized study data will be shared with any qualified investigator upon reasonable request.

### Statistical analysis

Statistical analysis was performed using SPSS 29 (IBM, CA, USA). For analysis of the time course of QMG scores, repeated measures-ANOVA was applied using the factor ‘ME’ (ME^+^ and ME^−^) and ‘age’ and ‘sex’ as covariates. Levene’s test was applied to check for homogeneity of variances. Greenhouse–Geisser correction was applied when the assumption of sphericity was violated. Post hoc tests compared QMG at the specific time points.

For comparison of secondary outcome parameters (steroid doses, escalation of medical therapy, hospitalization due to MG, comorbidities) of patients with and without ME, analysis of variance (ANOVA) was used. Kolmogorov–Smirnov tests of normality were performed for all data sets and parametric or non-parametric statistics were used where appropriate. Data are shown as median ± interquartile range (IQR) for non-parametric data or as mean ± standard error (SE) for parametric data. A *p*-value < 0.05 was considered significant.

## Results

### Baseline characteristics and clinical features

80 Patients were included (51 women, 29 men; mean age 57 ± 15 years). 60 patients were seropositive (76%) with antibodies detectable against acetylcholine receptors (AChR^+^; 56 patients), muscle-specific kinase (MuSK^+^: three patients), and lipoprotein receptor-related protein 4 (LRP4; one patient).

12 patients experienced a ME and 2 patients suffered from manifest MC. In ME^+^ patients, no provocation factors for ME could be noticed including infections or changes in medication.

Baseline epidemiologic criteria including sex and age among ME^+^ and ME^−^ patients appeared balanced including presence of MG-related antibodies (ME^+^: AChR^+^: nine patients; LRP4^+^: one patient; seronegative: four patients; ME^−^: AChR^+^: 47 patients; MuSK: three patients; seronegative: 16 patients; *p* = *0.94*) or disease duration (ME^+^: 56.4 months; ME^−^: 86.5 months, *F* = 0.7; *p* = 0.41).

20 patients previously underwent thymectomy (ME^+^: 6 patients; ME^−^: 14 patients; *p* = 0.44). Of those, 17 patients were AChR^+^ and three patients were seronegative. During our observation period, further four patients underwent thymectomy. Recognized thymoma were equally distributed among groups with 6/66 cases in ME- and 1/14 cases in ME^+^ patients (*p* = 0.28).

However, we found higher baseline impairment reflected by the MGFA-class at baseline in ME^+^ patients with ten patients experiencing baseline MGFA-class ≥ II (73%) compared to four ME^−^ patients (27%; *p* < 0.001). This is also reflected in higher QMG scores upon baseline as described below. In line with this, ME^+^ patients were treated significantly more often with immunotherapy upon first admission (ME^+^: *n* = 12; ME^−^: *n* = 9; *F* = 32.78; *p* < 0.001). Immunotherapy at baseline included azathioprine (ME^+^: five patients; ME^−^: seven patients) and mycophenolate (ME^+^: four patients; ME^−^: nil patient). Among ME^+^ patients, one patient received IVIgs, one received rituximab, and one received cyclophosphamide at baseline. Among ME^−^ patients, one patient received cyclosporine and another one received infliximab due to existing rheumatoid arthritis as well.

Also, a higher number of patients received steroids in the ME^+^ group (ME^+^: 8/14; ME^−^: 7/66 patients; *F* = 17.23; *p* < 0.001) and mean steroid dose was higher in the ME^+^ group (ME^+^: 13.9 mg ± 5.2; ME^−^ 1.2 mg ± 0.5; *F* = 27.4; *p* < 0.001*).*

Rescue treatment of ME comprised of PLEX in four patients, IVIg in five patients and five patients underwent subsequent treatment with PLEX and IVIg due to insufficient response. Five patients required ICU treatment; nine patients were admitted to intermediate care unit. Two patients required mechanical ventilation and of those, one patient experienced temporary cardiac arrest. In the other three patients admission to ICU was indicated due to clinical instability and need for intensive care to prevent from ventilation. In general, time to first outpatient visit of ME^+^ patients was 1 month [IQR 1–2 months].

Contrary to ME^−^ patients, only ME^+^ patients required an escalation of existing immunotherapy during follow-up because of subacute deterioration (ME^+^: 11/14 patients; ME: 0/66 patients; *F* = 125.1; *p* < 0.001), whereas ME^−^ patients remained stable throughout. Escalation medication comprised of recurrent IVIg (six patients), rituximab (one patient), ravulizumab (two patients), cyclophosphamide (one patient), or recurrent immunoadsorption (one patient).

Disease duration from symptom onset to start of immunotherapy appeared equally balanced among both groups (ME^+^: 3 months [IQR: 3–6]; ME^−^: 6 months [IQR: 1–23]; *p* = 0.89). Patients were started with first-line treatments (azathioprine, mycophenolate, or methotrexate) throughout in our cohort.

Regarding comorbidities, concomitant rheumatoid diseases were significant more frequent in ME^+^ patients (ME^+^: 3/14 patients (21%); ME^−^: 3/66 patients (5%); *F* = 4.2; *p* = 0.042).

Only ME^−^ patients were affected from thyroid diseases, however no significant difference could be found (ME^+^ 0/14 patients (0%), ME^−^ 5/66 patients (8%), *F* = 0.11, *p* = 0.7). For details see Table 1.

**Table 1:** depicts the clinical and demographic data of the included patients. Disease duration did not differ between the patient groups (*p* = 0.41). ME^+^ patients were more severely affected regarding MGFA class at entrance of the study (*F* = 85.6; *p* < 0.001), needed more intensive immunosuppressant therapy and were more frequently hospitalized even after ME as indications for higher disease activity. Concomitant rheumatoid diseases were significantly more frequent in ME^+^ patients (*F* = 4.2; *p* = 0.042)

	ME + (*n* = 14)	ME- (*n* = 66)	*p*-value
Age, yrs, mean (SD)	54.6 (17.2)	57.7 (14.7)	0.38
Male patients (%)	2 (14)	27 (41)	
Disease duration since diagnosis, mo, median (IQR)	56 (48–126)	87 (21–123)	0.41
Disease duration until MC, mo, median (IQR)	60 (34–119)	n/a	
MGFA class at baseline (%)			< 0.001
MGFA I	0 (0)	35 (53)	
MGFA II	4 (29)	27 (41)	
MGFA III	2 (14)	4 (6)	
MGFA IV	5 (36))	0	
MGFA V	3 (21)	0	
Antibody distribution			0.94
AChR + (%)	9 (64)	47 (71)	
MuSK + (%)	0 (0)	3 (5)	
LRP4 + (%)	1 (7)	0 (0)	
AChR- MuSK- (%)	4 (29)	16 (24)	
Underwent thymectomy (%)	6 (42)	14 (21)	0.44
Steroid treatment at baseline (%)	8 (57)	7 (11)	< 0.001
Steroid dose baseline, mg, mean (SD)	13.9 (5.2)	1,2 (0.5)	< 0.001
Immunotherapy baseline (%)	12 (86)	9 (14)	< 0.001
Azathioprine (%)	5 (36)	7 (11)	
Mycophenolate (%)	4 (29)	0	
Cyclosporine (%)	0	1 (2)	
Rituximab (%)	1 (7)	0	
Cyclophosphamide (%)	1 (7)	0	
Escalation of immunotherapy (%)	11 (79)	0	< 0.001
Hospitalization (%)	7 (50)	4 (6)	< 0.001
Concomitant autoimmune disease (%)	3 (21)	3 (5)	0.04
Thyroideal disease (%)	0(0)	5 (8)	0.7

### Outcome of MC

Following ME, QMG scores remained higher in ME^+^ patients following re-baselining compared to their ME^−^ counterparts. Notably, we found a slight but significant reduction of QMG scores also in ME^−^ patients following admission to our center which due to initiation or optimization of therapy disease severity improved in MC^−^ patients significantly after three months (*p* = 0.001) as well as 12 months (*p* = 0.049).

**Fig. 1:**
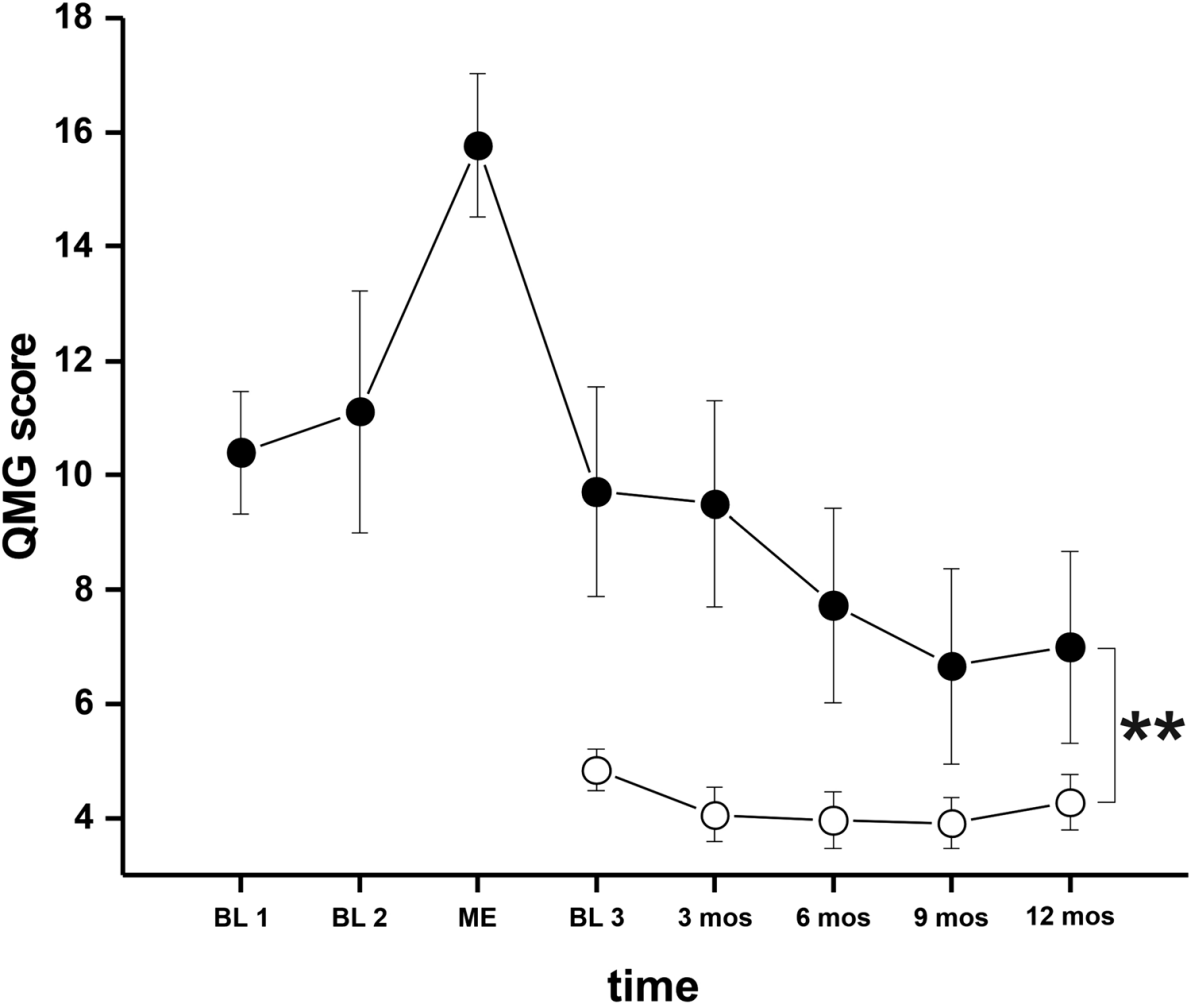
depicts the course of QMG during the observation period in ME^+^ (black squares) and ME^−^ (open squares) patients. Repeated measures ANOVA showed that the time course of QMG is significantly different between the two groups of patients (*F* = 8.2; *p* = 0.006). Further analysis revealed that QMG scores are significantly higher in ME^+^ patients at the first visit in the outpatient clinic after ME (*F* = 19, *p* < 0.001) as well as after three (*F* = 21.0, *p* < 0.001) and six months (*F* = 6.5, *p* = 0.013). At 9 months, there is a tendency for higher QMG scores in the ME^+^ group, however, level of significance is not met (*F* = 2.6, *p* = 0.072). At 12 months, no significant difference could be found (*F* = 2.3; *p* = 0.13). **p < 0.01

In more detail, QMG scores rapidly declined to pre-ME levels in ME^+−^patients following acute treatment. However, we observed only a modest further decrease of QMG scores during months 3–12 indicative of the previously described “therapeutic lag” of immunomodulatory treatment (*F* = 8.2; *p* = 0.006; Fig. 1).

Of note, QMG scores at “baseline 1&2” are substantially elevated compared to baseline levels of ME^−^ patients already and suggest poor disease control due to highly active MG prior ME and might thus explain the susceptibility of patients towards ME in absence of further trigger factors. One patient manifested with ME and thus, no QMG scores were available at “baseline 1&2”.

Hospitalization after baseline was necessary significant more frequent in ME^+^ patients than in ME^−^ (ME^+^: 7/14 patients (50%); ME^−^ 6/66 patients (9%); *F* = *14.5; p* < *0.001*).

## Discussion

We here evaluated whether the presence of ME affects the subsequent disease course in MG patients. We show that ME persistently worsens the disease course despite subsequent escalation of immunosuppressive therapy.

Thus, our results indicate that the prevention of ME might be crucial for the long-term clinical status of patients with MG. Our data argue for an early escalation of therapy in patients with a high burden of disease and indication of a highly active MG.

The ME group had higher QMG values before ME (besides the patient presenting with ME as first symptom as well as the patient with so far ocular MG until ME). Therefore, these patients have a high burden of disease and can be classified as highly active MG although immunotherapy existed in accordance with the guidelines. But, ME might be a preventable situation by early start and especially timely escalation of immunotherapy.

ME is characterized by severe clinical deterioration, need of hospitalization and appropriate therapy and is associated with high burden of disease [14]. Response to rescue therapy was consistent in our cohort irrespective of the chosen treatment and this in in line with the present literature [3]. Yet, response was restricted to alleviation towards baseline QMG scores in ME^+^ patients. In our cohort, ME^+^ patients remained substantially impaired throughout observation period despite escalation of immunosuppressive treatment and increase of corticosteroid doses.

Accordingly, additional hospitalization occurred more frequently in the ME^+^ group compared to the ME^−^ group. This finding is in line with the study of *Kalita *et al*.* [9]. Their patients with myasthenic crisis had more hospitalizations and consecutive consultations in the further course of the disease compared to patients without MC.

Huang et al. did not find a significant difference in the long-term outcome among MC^−^ and MC^+^ patients [8]. However, two thirds of their patients received no immunosuppressive therapy prior to acute dyspnea (and eventually subsequent ME/MC) and moreover, a substantial proportion of patients deteriorated in response to infections. Furthermore, patients received IVIg throughout. As MGFA scores prior to exacerbation were not mentioned, it remains unclear whether their cohort was generally better controlled compared to our ME^+^ patients.

Our patients were less severely affected by ME since only two patients required mechanical ventilation and only five patients required treatment in the intensive care unit. In the present study, ME was defined by need for rescue therapy, e.g. PLEX and IVIg.

Our results, along with other studies argue in favor of an early intervention to prevent the need of intensive care therapy and mechanical ventilation in ME [13].

Avoiding ME is a crucial factor to obtain a stable clinical status of patients with MG. As in previous studies [12, 15] patients with higher QMG scores reflecting active MG developed more frequently ME compared to patients with lower disease activity. Therefore, therapeutic control of disease activity appears to be crucial not only to prevent ME but for the severity of clinical impairment throughout the course of MG. This finding is relevant since there are new, very effective and well-tolerated drugs for MG approved, e.g. complement inhibitors and neonatal Fc-receptor (FcRn) modulators that are approved as add-on therapies in generalized MG [7, 10].

However, there are still other medications that have been used as escalation therapies in MG for a long time like cyclophosphamide or IVIg. In summary, an early escalation in the therapeutic strategy to achieve early control of disease activity appears to be beneficial for the patients. Escalation of therapy was performed in almost all ME^+^ patients. Therefore, 1 year after ME, the QMG score was decreased compared to the baseline reflecting an overall improvement of clinical status due to optimized therapy. However, patients did not recover to the clinical level of ME^−^ patients.

We found an apparent association of ME with rheumatoid disease, yet given the lower number of events, this might as well be an artefact.

### Limitations of the study

This is a post hoc analysis of a longitudinal monocenter cohort and thus faces the general limitations of such a study design. Additionally, our tertiary center faces a potential selection bias resulting in the high percentage of patients with exacerbations despite immunotherapy. However, the structured approach of our center towards these patients is reflected by the high availability of QMG scores as well as complete data on baseline parameters including thymectomy status and antibody testing. Additionally, our study does not comprise data on the “novel” substances used in MG treatment including FcRn modulators as well as complement inhibitors. However, our finding that higher QMG scores are associated with increased risks for ME remain independent from the type of escalation treatment.

## Conclusion

Patients with high QMG scores have an increased risk to develop ME since high QMG scores can be associated with high disease activity. Even after appropriate therapy of ME, this group of patients remains clinically more impaired indicating increased burden of disease and decreased quality of life. Therefore, early escalation of MG therapies in order to prevent ME might be beneficial for some patients.

## Data Availability

Entire data set is available upon reasonable request contacting A. Mück.

## Abbreviations

FcRn: :Neonatal Fc receptor
MC: :Myasthenic crisis
ME: :Myasthenic exacerbation
MGFA: :Myasthenia Gravis Foundation of America
AChR: :Acetylcholine receptor
MuSK: :Muscle-specific kinase
IVIg: :Immunoglobulin
